# Studies on Syngas Fermentation With *Clostridium carboxidivorans* in Stirred-Tank Reactors With Defined Gas Impurities

**DOI:** 10.3389/fmicb.2021.655390

**Published:** 2021-04-15

**Authors:** Anton Rückel, Jens Hannemann, Carolin Maierhofer, Alexander Fuchs, Dirk Weuster-Botz

**Affiliations:** Institute of Biochemical Engineering, Technical University of Munich, Garching, Germany

**Keywords:** *C. carboxidivorans*, continuously gassed stirred-tank bioreactor, syngas fermentation, gas impurities, inhibition studies

## Abstract

Syngas fermentation processes with acetogenic bacteria like *Clostridium carboxidivorans* have been proven to be a promising approach for the conversion of CO-rich waste gases into short- and medium-chain alcohols. The challenge of synthesis gas impurities, on the other hand, has always been a major concern for establishing an industrial-scale process, since some of the trace components in waste gases, such as NH_3_, H_2_S, and NO*_*x*_*, can have inhibiting or even toxic effects on microbial growth and product formation. Thus, this study aims to identify the effects of the main trace impurities in syngas from gasification of biogenic residues by the supply of defined concentrations of trace impurities to the cultivation medium. Autotrophic gas fermentation studies were performed with *C. carboxidivorans* in batch-operated fully-controlled stirred-tank bioreactors with continuous gas supply (80% CO and 20% CO_2_). The syngas components NH_3_ and H_2_S had a positive effect on both growth and alcohol formation (ethanol, 1-butanol, and 1-hexanol). The maximum biomass concentration was increased by more than 50%, and the maximum ethanol concentration was more than doubled with 5.0 g L^−1^ NH_4_Cl or 1.0 g L^−1^ H_2_S provided by the addition of 2.2 g L^−1^ thioacetamide. The addition of the nitrogen oxide species nitrate and nitrite, on the other hand, reduced biomass growth as well as alcohol concentrations. Already, the supply of 0.1 g L^−1^ NaNO_3_ resulted in reduced growth and 25% reduction of the maximum ethanol concentration. The production of the longer chain alcohols 1-butanol and 1-hexanol was reduced as well. All NaNO_2_ concentrations tested showed a strong toxic effect on the metabolism of *C. carboxidivorans*, and neither CO consumption nor product formation was observed after addition. As a consequence, NO*_*x*_* components in syngas from the gasification of biogenic residues should be reduced by the gasification process and/or selectively removed from the syngas after gasification.

## Introduction

Synthesis gas fermentation is an anaerobic process, which uses obligate anaerobic bacteria like *Clostridium* species, i.e., *Clostridium ljungdahlii*, *Clostridium autoethanogenum*, *Clostridium ragsdalei*, and *Clostridium carboxidivorans* to convert synthesis gas mixtures that mainly consist of H_2_, CO_2_, and CO into products like alcohols or other platform chemicals (Wilkins and Atiyeh, 2011). These so-called acetogenic bacteria use the reductive acetyl-CoA pathway, also known as Wood–Ljungdahl pathway, to convert gaseous C1 substrates into acetyl-CoA, and afterwards into a variety of C2–C8 products. The microorganism *C. carboxidivorans* is known for its ability to produce not only acetic acid and the short-chain alcohol ethanol, but also 1-butanol and 1-hexanol (Phillips et al., 2015; Fernández-Naveira et al., 2017), qualifying it as an interesting microorganism for industrial production (Daniell et al., 2012). The alcohols produced are of particular interest, because of their potential usage as fuels or fuel additives (Dürre, 2007). The pH optimum for growth is around pH 6.0, and pH controls the product spectrum of *C. carboxidivorans.* Reducing the pH < pH 6.0 triggers the alcohol formation. *C. carboxidivorans* typically shows a two-stage production behavior in batch processes consisting of an acidogenic phase, in which mainly organic acids are produced, and, after the decrease of pH due to acid formation, a solventogenic phase, during which the produced acids are reconsumed and converted into alcohols (Ukpong et al., 2012). *C. carboxidivorans* has a natural plasmid encoding genes for 15 hypothetical proteins with unknown function, three phage-related proteins, two integrase/recombinase proteins, and two proteins involved in chromosome partitioning, but neither of these were determined to be essential (Bruant et al., 2010; Li et al., 2015). Other *Clostridia* like *C. acetobutylicum* are known to have the genes encoding their proteins for butanol production on their plasmid (Cornillot et al., 1997). The qualification of *C. carboxidivorans* for the production in industrial reactor types has been shown for continuous stirred-tank bioreactors in series (Abubackar et al., 2018; Doll et al., 2018) as well as in bubble columns and trickle-bed reactors (Riegler et al., 2019). *C. carboxidivorans* has a wide tolerance for different CO partial pressures in the range from 0.35 to 2.0 bar (Hurst and Lewis, 2010).

A real synthesis gas, for example, from the gasification of biogenic residues, often consists of a wide variety of other components besides the main components CO, CO_2_, and H_2_ like ammonia, hydrogen cyanide, nitrogen oxide species, hydrogen sulfide, sulfoxides, COS, CS_2_, or short-chain hydrocarbons and tar (Briesemeister et al., 2017; Kremling et al., 2017). Especially the usage of synthesis gas from the gasification of biogenic residues is a promising approach for the application of a waste to value process. Typical orders of magnitude for the trace components in syngases from entrained-flow gasification of biogenic residues are 0.1–6.0% CH_4_, 4,500 ppm NH_3_, 150 ppm HCN, 200 ppm H_2_S, and 200 ppm NO*_*X*_* (Briesemeister et al., 2017; Kremling et al., 2017; Kremling, 2018). Some of these species can have beneficial effects on the microbial growth and production, while others can have inhibiting or even toxic effects.

Although studies have been performed that focused on the usage of real, biomass-derived synthesis gases, currently few studies have investigated the influences of the individual trace components to give further insight into the role of certain trace components (Datar et al., 2004; Ahmed et al., 2006; Xu et al., 2011; Infantes et al., 2020). The toxicity of HCN on the anaerobic bacteria *C. ljungdahlii* has already been analyzed and identified as a critical factor for the growth of anaerobic microorganisms with inhibiting effects at 1.6 mg L^−1^ and a strong toxic effect at concentrations of 0.065 g L^−1^ KCN in anaerobic shanking flasks (Oswald et al., 2018). The usage of NO_3_^–^ as an alternative terminal electron acceptor instead of CO or CO_2_ in the acetogenic metabolism of some *Clostridia* has been shown for *C. ljungdahlii* and represents a thermodynamically favorable mechanism for ATP conservation in the anaerobic bacteria with both increased growth and carbon dioxide fixation at NO_3_^–^ concentrations of 1.27 g L^−1^ of NaNO_3_ (Emerson et al., 2019; Klask et al., 2020). Energy conservation is a crucial aspect for growth and product formation in acetogenic bacteria, because the CO_2_-reducing metabolism does not lead to a net ATP gain (Schuchmann and Müller, 2014). The conversion of NO_3_^–^ has also been shown for the thermophilic anaerobic bacteria *Moorella thermoacetica* and *Clostridium thermoautotrophicum*, where even the usage of NO_2_^–^ was feasible in *M. thermoacetica* with concentrations up to 0.35 g L^−1^ NaNO_2_ (Seifritz et al., 1993, 2003; Fröstl et al., 1996; Arendsen et al., 1999).

Since the solved syngas impurities affect the metabolism of microorganisms, syngas impurities were added as their respective salt components. NH_4_Cl was used for studying NH_3_ impurities in syngas. Thioacetamide was used to generate H_2_S *in situ* at pH < 7 in the medium (Gunning, 1955). The *in situ* formation of H_2_S through thioacetamide always creates 1 mol of acetate and 1 mol of NH_4_^+^ for every 1 mol of H_2_S. Since the physical solubility of NO*_*x*_* is very low compared to its respective anionic forms NO_3_^–^ and NO_2_^–^ (Chan et al., 1976; Lee and Schwartz, 1981; Joshi et al., 1985), NaNO_3_ and NaNO_2_ were used to study the effects of dissolved NO*_*x*_* components on the metabolism of microorganisms.

This study deals with the influence of the individual trace gas components NH_3_, H_2_S, NO_3_^–^, and NO_2_^–^ on growth, product formation, and CO consumption of *C. carboxidivorans* in batch processes in continuously gassed stirred-tank bioreactors with artificial syngas as the sole energy and carbon source. These inhibition studies are used to define adequate concentration ranges for the trace components in a real synthesis gas, which can be used for the quantitative definition of syngas purification criteria.

This study aims to close the gap in the existing literature concerning the effects of individual trace impurities in syngases. Studies so far focused solely on the effects of syngas from gasification of biogenic residues and found inhibiting effects without clearly identifying the components responsible for the effects (Infantes et al., 2020; Liakakou et al., 2021). Also, most studies focused on *C. ljungdahlii* as a typical model organism for acetogenic bacteria, while this study used *C. carboxidivorans* as a promising acetogen for the production of longer chain alcohols.

## Materials and Methods

### Microorganisms and Cultivation Media

*Clostridium carboxidivorans* P7^T^ (DSM 15243) was obtained from the German Collection of Microorganisms and Cell Cultures GmbH (DSMZ GmbH, Braunschweig, Germany). The cultivation medium was described in a previous study (Doll et al., 2018). The detailed composition of the cultivation medium can be found in the Supplementary Material.

### Preculture Conditions in Shaken Anaerobic Bottles

Stocks of frozen cell broth at approximately 0.5 g L^−1^ cell dry weight (CDW) were kept in Hungate type tubes at −80°C with 10% glycerol as a protecting agent. A sample of 2.5 mL of the frozen cell broth was inoculated to an anaerobic bottle with 100 mL of the described cultivation medium with 5 g L^−1^ glucose and 15 g L^−1^ MES as a pH-buffer at pH 6.0. The headspace gas composition was approximately 5% H_2_ and 95% N_2_ at a total pressure of 1.0 bar to keep a reducing atmosphere in the anaerobic bottle. The anaerobic bottles were then incubated at 37°C and agitated at 100 min^−1^ in a WiseCube WIS-20 (Witeg Labortechnik GmbH, Wertheim, Germany) incubator. After 22 h, the culture was harvested and centrifuged anaerobically for 10 min at 3,600 rcf in a Rotixa 50 RS (Hettich GmbH & Co., KG, Tuttlingen, Germany). Afterward, the pellet was resuspended in 10 mL of anaerobic phosphate buffer (PBS) and used to inoculate the stirred-tank bioreactor with an initial biomass concentration of 0.05 g L^−1^ CDW.

### Continuously Gassed Stirred-Tank Bioreactor Setup for Syngas Fermentation Processes

All the experiments in this study were performed as batch processes in a continuously gassed stirred-tank bioreactor KLF2000 (BioEngineering AG, Wald, Switzerland) with a total volume of 2.4 L (*d*_tank_ = 98 mm), a working volume of 1.0 L at an agitation rate of 1,200 min^−1^ (volumetric power input P V^−1^ = 15.1 W L^−1^) with two six-blade rushton turbines (*d*_stirrer_ = 40 mm), a temperature of 37°C, an absolute pressure of 1.0 bar, and an initial pH 6.0. The reactor was sterilized in place at 121°C for 20 min with distilled water and at an agitation rate of 200 min^−1^. The cultivation medium was separately autoclaved in a 1-L flask with a butyl-rubber septum at 121°C for 20 min without the vitamins and cystein. The sterilized medium was transferred to the reactor using a peristaltic pump (Watson Marlow, Cornwall, England) and a sterile silicone tube. The cystein and vitamin solutions (see Supplementary Material) were sterilized by filtration through a 0.2-μm syringe filter and added to the cultivation media after the medium was cooled down below 40°C.

The medium in the reactor was anaerobized for approximately 2 h by sparging with N_2_ and afterward equilibrated with the syngas mixture for least 12 h. The gas supply was controlled with thermal mass flow controllers and mass flow meters Bronkhorst F-101D-RAD-33-V (Wagner Mess- und Regeltechnik, Offenbach, Germany). The flow rates of the syngas components were 4.0 L h^−1^ CO (*p*_CO_ = 800 mbar) and 1.0 L h^−1^ CO_2_ (*p*_CO__2_ = 200 mbar) for the processes in this study, if not mentioned differently.

Redox potential and the pH were measured during the fermentation process using pH and redox probes (405-DPAS-SC-K8S/120 and Pi 4805-SC-DPAS-K8S/120, Mettler Toledo, Gießen, Germany). The pH of the medium was adjusted to pH 6.0 with 3 M NaOH before inoculation. The pH of the batch processes was controlled with 1 M H_2_SO_4_, if a pH > 6.0 was measured. Redox potential was not controlled but monitored to ensure that anaerobic conditions (redox potential ≤ 200 mV) were always ensured in the batch processes.

The reference batch process was reproduced four times (including previously published data from our group, Doll et al., 2018), and the standard deviation is represented as error bars in the diagrams and is listed in the tables. All processes with defined syngas impurities were performed once, thus no error indicators are given for the experimental data obtained with these processes. The measurements of CDW and product concentrations were conducted in technical triplicates, which gave minimal standard deviations for all measurements, and were therefore not used as an error indicator.

### Supply of Defined Impurities

All the defined syngas impurity components were added to the reaction medium as stock solutions of a solid component. The chemicals used were NH_4_Cl, NaNO_3_, NaNO_2_, and thioacetamide with concentrations in the stock solution of 100, 10, 50, and 44 g L^−1^, respectively, in anaerobic distilled water. The pH of the thioacetamide solution was adjusted to pH 10 with 0.1 M anaerobic NaOH solution to avoid the dissociation of thioacetamide in the stock solution. NH_4_Cl, NaNO_3_, and thioacetamide were supplied immediately before the reactor was inoculated, while NaNO_2_ was added as a pulse after the CDW in the bioreactor had already reached more than 0.3 g L^−1^ CDW. The concentrations of the trace components in the reactor were chosen individually based on pre-studies in shaken anaerobic bottles (data not shown). These were 10.0, 7.5, and 5.0 g L^−1^ NH_4_Cl, respectively. The concentrations of thioacetamide were calculated according to the expected production of H_2_S assuming total conversion of the thioacetamide added to achieve 2.0, 1.0, and 0.1 g L^−1^ H_2_S, respectively. The concentrations of NaNO_3_ in the reactor were adjusted to 2.0, 1.0, and 0.1 g L^−1^ NaNO_3_, respectively. The NaNO_2_ concentrations after the stock solution was added were 0.5, and 0.1 g L^−1^, respectively.

### Analytical Methods

#### Liquid Product Analysis

All the liquid samples were collected by a sampling valve at the bottom of the bioreactor and used to analyze biomass and product concentration. The samples were analyzed with a UV–Vis spectrophotometer (Genesys 10S UV–Vis, Thermo Scientific, Neuss, Germany) for optical density (OD_600__nm_) measurements, and a correlation factor was used to obtain CDW concentrations. Maximum specific growth rates were estimated by applying a non-linear regression to the experimentally obtained data for CDW concentrations. High-performance liquid chromatography (HPLC; Finnigan Surveyor Plus, Thermo Scientific, Waltham, MA, United States) was used to analyze the product and metabolite concentrations in the liquid phase (formic acid, acetic acid, butyric acid, caproic acid, ethanol, and 1-butanol). The samples with 1-hexanol were extracted and concentrated with ethyl acetate in a ratio of 3:1 (sample to solvent) before the measurement. The HPLC instrument used a refractive index detector to detect the products and a cation exchange separation column HPX-87H (Bio-Rad, Munich, Germany) at a column temperature of 60°C. The elution was carried out isocratically with 5 mM H_2_SO_4_ at a flow rate of 0.6 mL min^−1^.

#### Exhaust Gas Analysis

The exhaust gas of the bioreactor was analyzed online using a mass flow meter (Wagner Mess- und Regeltechnik, Offenbach, Germany) and a micro gas chromatograph (μGC 490, Agilent Technologies, Waldbronn, Germany). The gas components CO, CO_2_, and H_2_ were separated with a 1 m COX HI BF separation column at 2.5 bar and a column temperature of 80°C with N_2_ as the carrier gas, and measured by a thermal conductivity detector as previously described (Doll et al., 2018). Based on the gas flow rate measured online by the thermal mass flow meter and the gas partial pressure measured online by the μGC with a step-time of 10 min, the volumetric gas uptake or production rates were estimated for the gas components CO, CO_2_, and H_2_.

## Results

**FIGURE 1:**
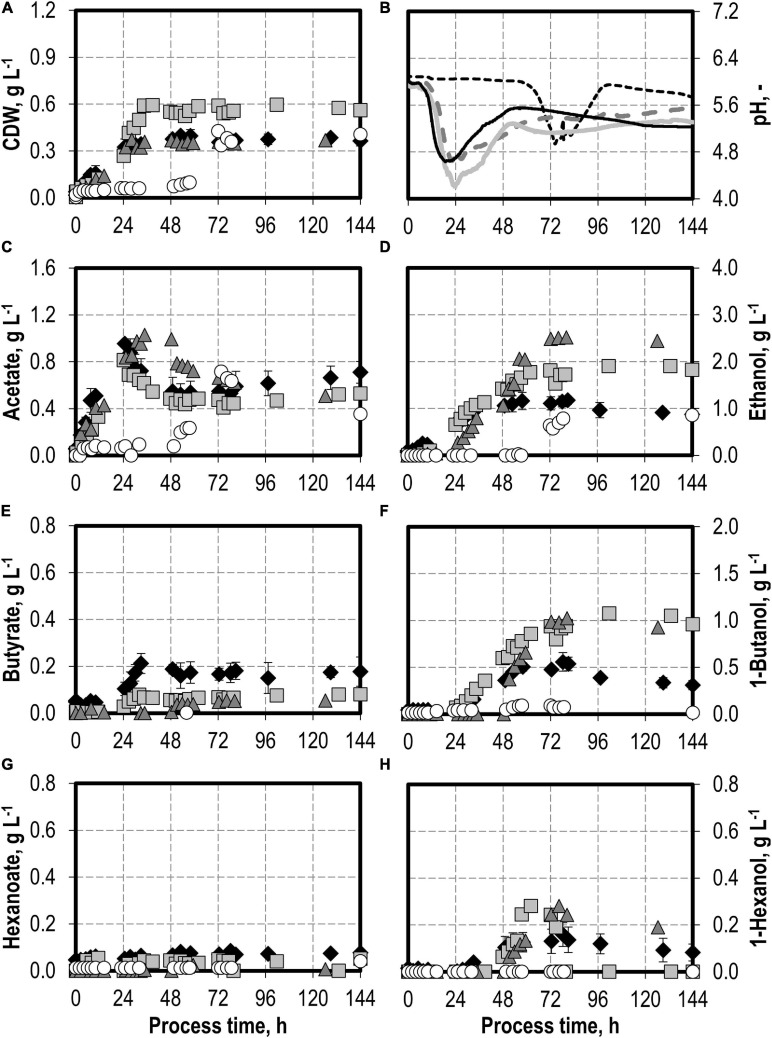
Autotrophic batch processes in continuously gassed stirred-tank bioreactors with *C. carboxidivorans* (*p*_CO_ = 800 mbar, *p*_CO__2_ = 200 mbar, *F*_gas_ = 0.083 L L^−1^ min^−1^, *T* = 37°C, P V^−1^ = 15.1 W L^−1^, pH_ini_ = 6.0, no pH control) with no initial addition of NH_4_Cl (reference process, black diamond, black line), addition of 5.0 g L^−1^ NH_4_Cl (gray square, gray solid line), addition of 7.5 g L^−1^ NH_4_Cl (gray triangle, gray dashed line), and addition of 10.0 g L^−1^ NH_4_Cl (white circle, gray dotted line). Error bars indicate the standard deviation of four reference processes; the other batch processes were performed once.

First, a batch process was carried out with *C. carboxidivorans* without any syngas impurities as reference. The reference process reached a maximum of 0.40 g L^−1^ CDW after 53 h and 0.96 g L^−1^ acetate after 24.75 h, combined with the pH reduction from pH 6.0 to pH 4.6 with a later reduction of the acetate to 0.52 g L^−1^ after 53 h and a pH increase to pH 5.5 (see Figures 1A,B). The alcohol production reached a maximum of 1.17 g L^−1^ ethanol, 0.56 g L^−1^ butanol, and 0.16 g L^−1^ hexanol, respectively (see Figures 1D,F,H). This is within the estimation error of the previously published results (1.21 g L^−1^ ethanol, 0.44 g L^−1^ butanol, and 0.15 g L^−1^ hexanol, respectively; Doll et al., 2018).

### Autotrophic Batch Processes With Varying Initial Ammonium Additions

The effects of varying initial ammonium additions on batch process performances of *C. carboxidivorans* are shown in Figure 1. The initial addition of 5.0 g L^−1^ NH_4_Cl resulted in an increase in the maximum CDW concentration by 50% compared to the reference process to 0.60 g L^−1^ after 34.5 h. Minimum pH was lower (pH 4.2 after 23.5 h) compared to the reference process (see Figure 1B). Higher alcohol concentrations were observed at the maximum (1.91 g L^−1^ ethanol, 1.08 g L^−1^ butanol, and 0.28 g L^−1^ hexanol). The accumulation of organic acids (see Figures 1C,E,G) was very low compared to the reference process, and the re-consumption of acetic acid led to a reduced final concentration of 0.53 g L^−1^ acetate. Longer chain carboxylic acids were almost entirely re-consumed (final concentrations: 0.08 g L^−1^ butyrate and 0.05 g L^−1^ hexanoate, respectively).

Increasing the initial ammonium concentrations further did not result in increased final CDW concentrations compared to the reference process with *C. carboxidivorans*. The initial addition of 10.0 g L^−1^ NH_4_Cl resulted in a drastically prolonged lag phase with almost no growth of *C. carboxidivorans* within 57.5 h after inoculation (Figure 1). As a consequence, the pH drop due to the formation of acids was delayed as well and the formation of C4 and C6 products was drastically reduced. The maximum ethanol concentration was highest with the initial addition of 7.5 g L^−1^ NH_4_Cl (2.52 g L^−1^ ethanol after 80 h), and the maximum butanol and hexanol concentrations were improved compared to the reference batch process (1.02 g L^−1^ butanol and 0.28 g L^−1^ hexanol).

**FIGURE 2:**
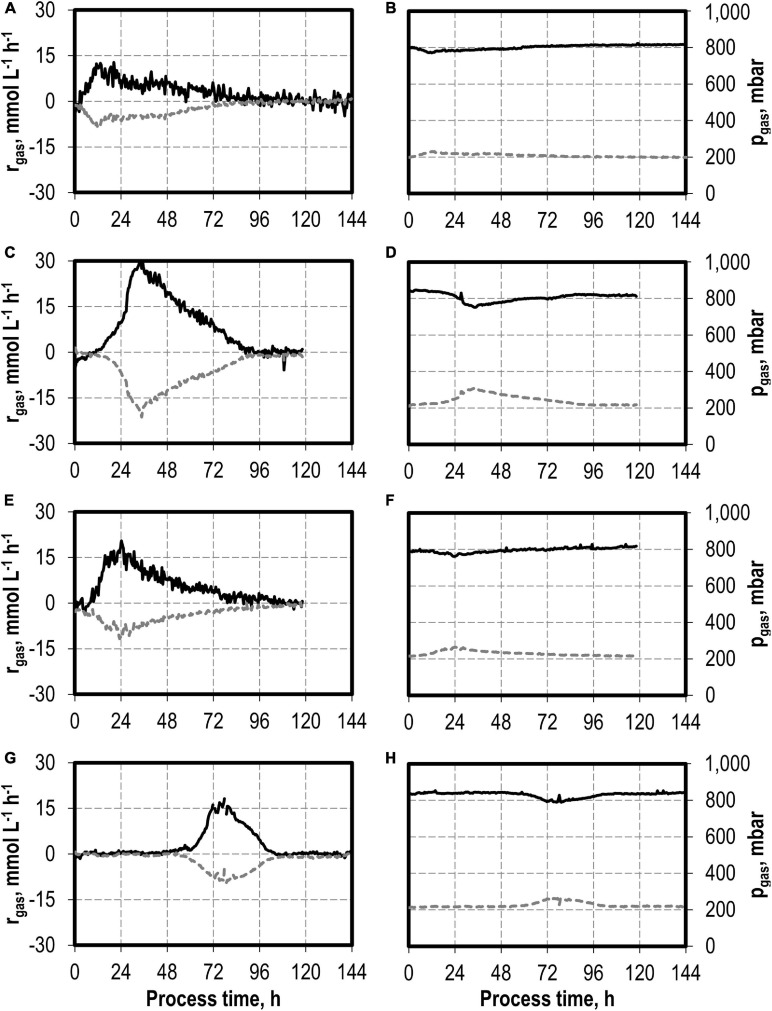
Gas consumption rates and gas partial pressures of CO (black line) and CO_2_ (dark gray dotted line) at autotrophic batch processes in continuously gassed stirred-tank bioreactors with *C. carboxidivorans* (*p*_CO_ = 800 mbar, *p*_CO__2_ = 200 mbar, *F*_gas_ = 0.083 L L^−1^ min^−1^, *T* = 37°C, P V^−1^ = 15.1 W L^−1^, pH_ini_ = 6.0, no pH control) with no initial addition of NH_4_Cl **(A,B)**, addition of 5.0 g L^−1^ NH_4_Cl **(C,D)**, addition of 7.5 g L^−1^ NH_4_Cl **(E,F),** and addition of 10.0 g L^−1^ NH_4_Cl **(G,H)**.

The maxima of the CO consumption and CO_2_ production rates were highest with the initial addition of 5.0 g L^−1^ NH_4_Cl, followed by initial 7.5 and 10 g L^−1^ NH_4_Cl, whereas the reference process showed the lowest maxima (Figure 2). The partial pressures in the reactors were only slightly reduced compared to the inlet partial pressures, due to the high gas flow rates in the batch processes (Figure 2). In combination with the high-power input, non-C-limiting process conditions can be assumed in all batch processes.

The total consumption of CO and production of CO_2_ of the batch processes are summarized in Table 1 on a C-mol basis. CO consumption was highest in the autotrophic batch process with the initial addition of 5.0 g L^−1^ NH_4_Cl (1,002.58 mmol CO—increase by a factor of 2.3 compared to the reference process), and the initial addition of 7.5 g L^−1^ NH_4_Cl resulted in an increase of 45% (632.38 mmol CO) compared to the reference. The CO/CO_2_ ratio of all batch processes was around 1.4, with exception of the reference process (1.33). Carbon recovery varies between 86 and 95%.

**TABLE 1:** Total consumption of CO and production of CO_2_, biomass, and products on a C-mol basis and carbon balances, as well as maximum CDW concentrations, maximum product concentrations, and the maximum specific growth rate of the autotrophic batch processes with *C. carboxidivorans* in continuously gassed stirred-tank bioreactors (*V* = 1 L) with the initial supplementation of NH_4_Cl.

	Reference process	+5.0 g L^−1^ NH_4_Cl	+7.5 g L^−1^ NH_4_Cl	+10.0 g L^−1^ NH_4_Cl
Carbon in medium, mmol C	9.94	9.94	9.94	9.94
CO consumption, mmol C	436.69 ± 5.7	1,002.58	632.38	411.86
CO_2_ production, mmol C	328.11 ± 14.5	720.22	450.54	281.41
CO:CO_2_ ratio, –	1.33 ± 0.06	1.39	1.40	1.40
Carbon in products, mmol C	81.06 ± 1.03	152.86	123.26	51.14
Carbon in biomass, mmol C	13.98 ± 0.34	21.49	14.14	15.58
C-balance (recovery), %	95 ± 3.0%	88%	92%	86%
CDW_max_, g L^−1^	0.40 ± 0.04	0.60	0.37	0.43
c_Acetate, max_, g L^−1^	0.96 ± 0.03	0.82	1.03	0.72
c_Ethanol, max_, g L^−1^	1.17 ± 0.18	1.91	2.52	0.86
c_Butyrat__, max_, g L^−1^	0.21 ± 0.04	0.08	0.05	0.00
c_1__–__But__anol__, max_, g L^−1^	0.56 ± 0.10	1.08	1.02	0.09
c_Hexanoate, max_, g L^−1^	0.08 ± 0.01	0.05	0.02	0.04
c_1__–Hexanol, max_, g L^−1^	0.16 ± 0.04	0.28	0.28	0.00
μ_max_, h^−1^	0.116 ± 0.008	0.162	0.114	0.058

*Standard deviations are estimated based on the results of four individual reference processes; other batch processes were performed once.*

Carbon balances are not fully closed because non-consumed organic carbon in the medium (yeast extract and cystein) and evaporation of the alcohols produced were not considered.

### Autotrophic Batch Processes With Varying Initial Thioacetamide (H_2_S) Additions

**FIGURE 3:**
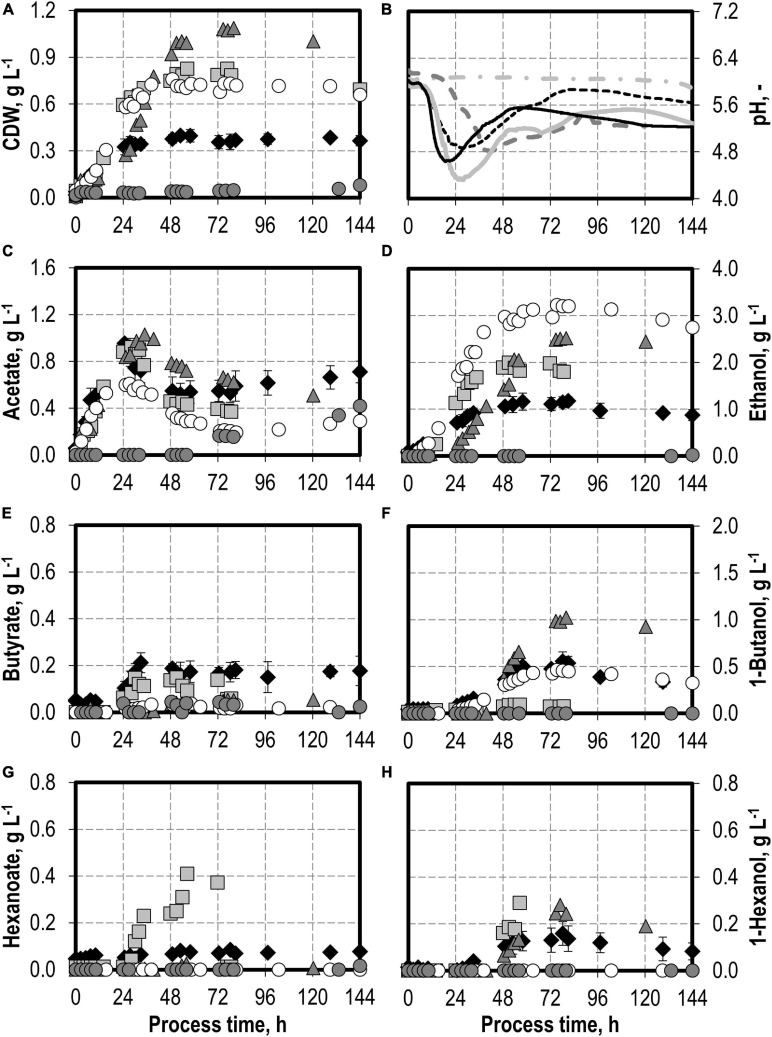
Autotrophic batch processes in continuously gassed stirred-tank bioreactors with *C. carboxidivorans* (*p*_CO_ = 800 mbar, *p*_CO__2_ = 200 mbar, *F*_gas_ = 0.083 L L^−1^ min^−1^, *T* = 37°C, P V^−1^ = 15.1 W L^−1^, pH_ini_ = 6.0, no pH control) with no addition of H_2_S (reference process, black diamond, black line), initial addition of 0.1 g L^−1^ H_2_S (gray square, gray solid line), initial addition of 1.0 g L^−1^ H_2_S (gray triangle, gray dashed line), initial addition of 0.5 g L^−1^ H_2_S with *p*_CO_ = 600 mbar, *p*_CO__2_ = 200 mbar, and *p*_H__2_ = 200 mbar (white circle, gray dotted line), and initial addition of 2.0 g L^−1^ H_2_S (gray circle, gray dashed–dotted line). Error bars indicate the standard deviation of four reference processes; other batch processes were performed once.

The effects of varying initial hydrogen sulfide concentrations in the medium are shown in Figure 3. The initial addition of 0.1 g L^−1^ H_2_S (0.22 g L^−1^ thioacetamide) or 0.5 g L^−1^ H_2_S (1.1 g L^−1^ thioacetamide) enabled improved growth of *C. carboxidivorans* to a maximum of 0.76 g L^−1^ CDW. The biomass concentration was more than doubled compared to the reference batch process (see Figure 3). The highest CDW concentrations of 1.09 g L^−1^ were observed after the initial addition of 1.0 g L^−1^ H_2_S (2.2 g L^−1^ thioacetamide), but the lag phase was prolonged considerably. The initial addition of 2.0 g L^−1^ H_2_S (4.4 g L^−1^ thioacetamide) prolonged the lag phase further to 132 h. However, after 244 h, the same CDW concentrations were achieved as after the initial addition of 1.0 g L^−1^ H_2_S (data not shown).

Hexanoate and hexanol concentrations were highest in the batch process with 0.1 g L^−1^ H_2_S (0.38 g L^−1^ hexanoate represents an increase by a factor of 4.75 and 0.28 g L^−1^ hexanol represents an increase by a factor of 1.8 compared to the reference process). Ethanol production was highest with the initial addition of 0.5 g L^−1^ H_2_S. A maximum concentration of 3.23 g L^−1^ ethanol was measured (increase by a factor of three compared to the reference process). The highest butanol concentration of 1.02 g L^−1^ was observed with the initial addition of 1.0 g L^−1^ H_2_S (increase by a factor of two compared to the reference process). A further increase in the maximum ethanol (3.59 g L^−1^) and butanol (1.09 g L^−1^) concentrations was measured after 244 h in the batch process with 2.0 g L^−1^ H_2_S (data not shown). However, no production of hexanol was observed in this process.

**FIGURE 4:**
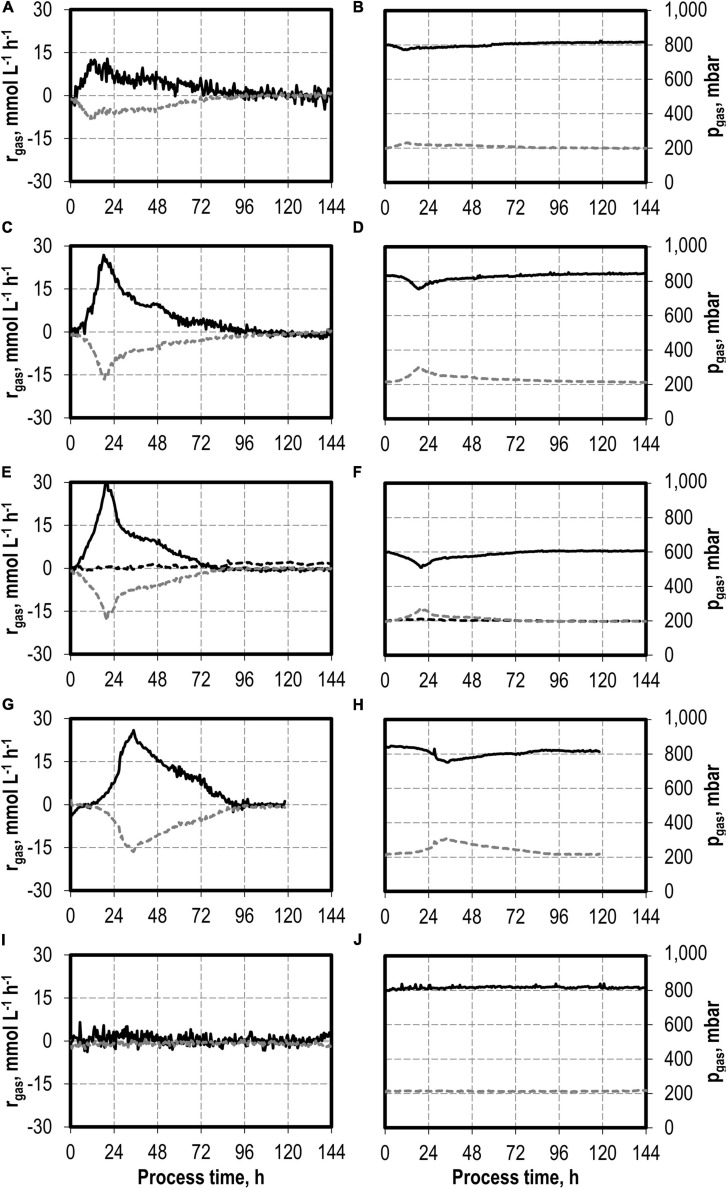
Gas consumption rates and gas partial pressures of CO (black solid line), CO_2_ (dark gray dotted line), and H_2_ (black dotted line) at autotrophic batch processes in continuously gassed stirred-tank bioreactors with *C. carboxidivorans* (*p*_CO_ = 800 mbar, *p*_CO__2_ = 200 mbar, *F*_gas_ = 0.083 L L^−1^ min^−1^, T = 37°C, P V^−1^ = 15.1 W L^−1^, pH_ini_ = 6.0, no pH control) with no addition of H_2_S **(A,B)**, initial addition of 0.1 g L^−1^ H_2_S **(C,D)**, initial addition of 0.5 g L^−1^ H_2_S with *p*_CO_ = 600 mbar, *p*_CO__2_ = 200 mbar, and p_H__2_ = 200 mbar **(E,F)**, initial addition of 1.0 g L^−1^ H_2_S **(G,H),** and initial addition of 2.0 g L^−1^ H_2_S **(I,J)**.

In accordance with the increased biomass and product concentrations observed with the initial addition of 0.1–1.0 g L^−1^ H_2_S, higher maxima of the CO consumption and CO_2_ production rates were measured compared to the reference batch process with *C. carboxidivorans* (Figure 4). In the process with 0.5 g L^−1^ H_2_S, the gas composition was slightly modified (600 mbar CO, 200 mbar CO_2_, and 200 mbar H_2_) compared to the other batch processes (800 mbar CO and 200 mbar CO_2_). This modification should reflect more closely the gas composition of a real syngas from gasification of biogenic residues. No consumption of the added H_2_ could be detected within the estimation error.

The maximum CO consumption rate was 31.6 mmol CO L^−1^ h^−1^ in the process with 0.5 g L^−1^ H_2_S, representing the highest increase in CO consumption rate in this study. Despite the increased metabolic activities of *C. carboxidivorans* caused by the initial H_2_S additions, the partial pressures in the reactors were only slightly reduced compared to the inlet partial pressures (Figure 4). In combination with the high power input, non-CO-limiting process conditions can be assumed in all batch processes.

The total consumption of CO and production of CO_2_ increases with increasing initial H_2_S additions compared to the reference batch process by 47% (0.1 g L^−1^ H_2_S), 62% (0.5 g L^−1^ H_2_S), 88% (1.0 g L^−1^ H_2_S), and by a factor of 2.9 (2.0 g L^−1^ H_2_S), respectively (Table 2). The CO/CO_2_ ratio of all batch processes was between 1.39 and 1.47 with the exception of the reference process (1.33). Carbon recovery varies between 87 and 100%.

**TABLE 2:** Total consumption of CO and production of CO_2_, biomass, and products on a C-mol basis and carbon balances, as well as maximum CDW concentrations, maximum product concentrations, and the maximum specific growth rate of the autotrophic batch processes with *C. carboxidivorans* in continuously gassed stirred-tank bioreactors (*V* = 1 L) with supplementation of thioacetamide (H_2_S).

	Reference process	+0.1 g L^−1^ H_2_S	+0.5 g L^−1^ H_2_S	+1.0 g L^−1^ H_2_S	+2.0 g L^−1^ H_2_S
Carbon in medium, mmol C	9.94	12.61	23.25	36.56	63.19
CO consumption, mmol C	436.69 ± 5.7	706.38	707.54	821.99	1,287.83
CO_2_ production, mmol C	328.11 ± 14.5	480.95	488.34	587.77	924.25
CO:CO_2_ ratio, –	1.33 ± 0.06	1.47	1.45	1.40	1.39
Carbon in products, mmol C	81.06 ± 1.03	205.53	138.07	159.14	215.16
Carbon in biomass, mmol C	13.98 ± 0.34	31.68	41.11	28.07	30.59
C-balance (recovery), %	95 ± 3.0%	100%	91%	90%	87%
CDW_max_, g L^−1^	0.40 ± 0.04	0.83	1.09	0.76	0.92
c_Acetate, max_, g L^−1^	0.96 ± 0.03	0.92	1.03	0.61	0.70
c_Ethanol, max_, g L^−1^	1.17 ± 0.18	2.00	2.52	3.23	2.68
c_Butyrat, max_, g L^−1^	0.21 ± 0.04	0.15	0.05	0.20	0.05
c_1__–Butanol, max_, g L^−1^	0.56 ± 0.10	0.09	1.02	0.46	0.59
c_Hexanoate, max_, g L^−1^	0.08 ± 0.01	0.41	0.02	0.00	0.02
c_1__–Hexanol, max_, g L^−1^	0.16 ± 0.04	0.29	0.28	0.00	0.00
μ_max_, h^−1^	0.116 ± 0.008	0.135	0.131	0.085	0.069

*Standard deviations are estimated based on the results of four individual reference batch processes; other batch processes were performed once.*

Carbon balances are not fully closed because non-consumed organic carbon in medium (yeast extract, cystein, and thioacetamide) and evaporation of the alcohols produced were not considered.

### Autotrophic Batch Processes With Varying Nitrate Additions

**FIGURE 5:**
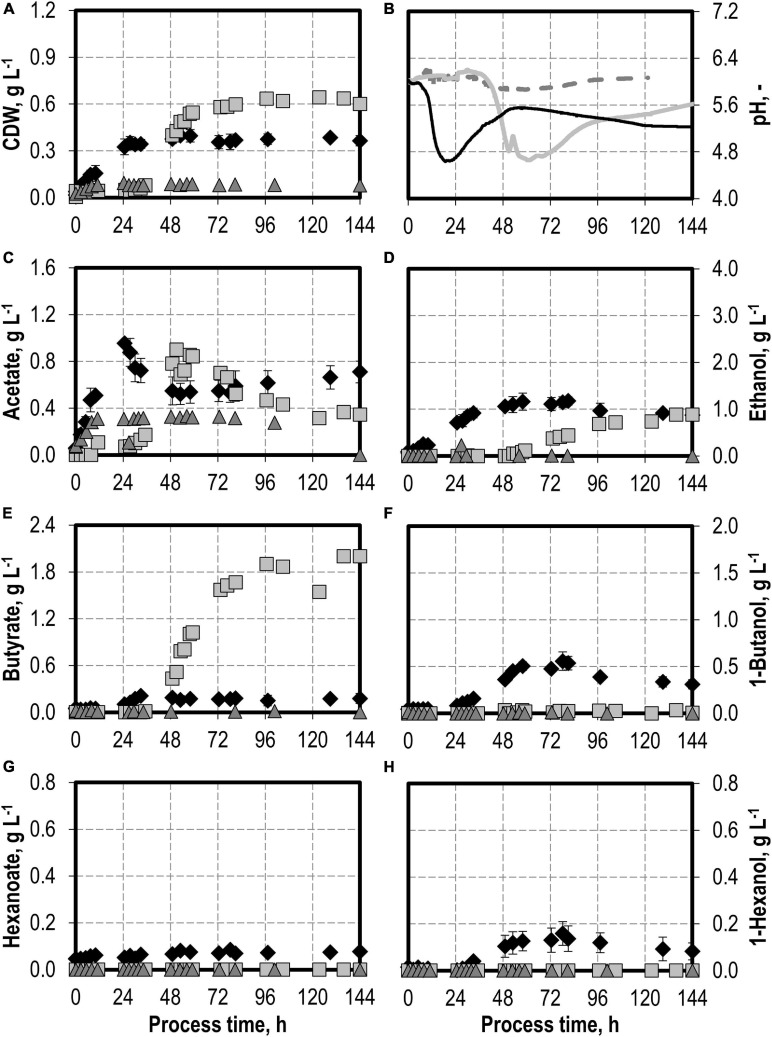
Autotrophic batch processes in continuously gassed stirred-tank bioreactors with *C. carboxidivorans* (*p*_CO,ini_ = 800 mbar, *p*_CO__2__,ini_ = 200 mbar, *F*_gas_ = 0.083 L L^−1^ min^−1^, *T* = 37°C, P V^−1^ = 15.1 W L^−1^, pH_ini_ = 6.0, no pH control below pH 6.0 but one-sided control above pH 6.1) with no addition of NaNO_3_ (reference process, black diamond, black line), initial addition of 0.1 g L^−1^ NaNO_3_ (gray square, gray solid line), and initial addition of 1.0 g L^−1^ NaNO_3_ (gray triangle, gray dashed line). Error bars indicate the standard deviation of four reference processes**;** other batch processes were performed once.

The effects of varying nitrate concentrations in the medium on batch process performances of *C. carboxidivorans* are shown in Figure 5. The initial addition of 0.1 g L^−1^ NaNO_3_ increased the biomass concentration by 60% compared to the reference process but with a considerably prolonged lag phase of 30 h. During the lag phase, pH was controlled with anaerobic 1 M H_2_SO_4_ to avoid a pH increase above pH 6.1. The maximum concentrations of the C2 products were slightly reduced compared to the reference process (0.90 g L^−1^ acetate was a reduction of 6% and 0.88 g L^−1^ ethanol was a reduction of 25%). The butyrate concentration, on the other hand, was increased drastically to 2.04 g L^−1^ butyrate after 144 h (increase by a factor of 9.7 compared to the reference process). All the other products, namely, butanol, hexanoate, and hexanol, were below the detection limit. Initial additions of higher nitrate amounts (1.0 g L^−1^) inhibited the growth of *C. carboxidivorans* and product formation drastically (Figure 5).

**FIGURE 6:**
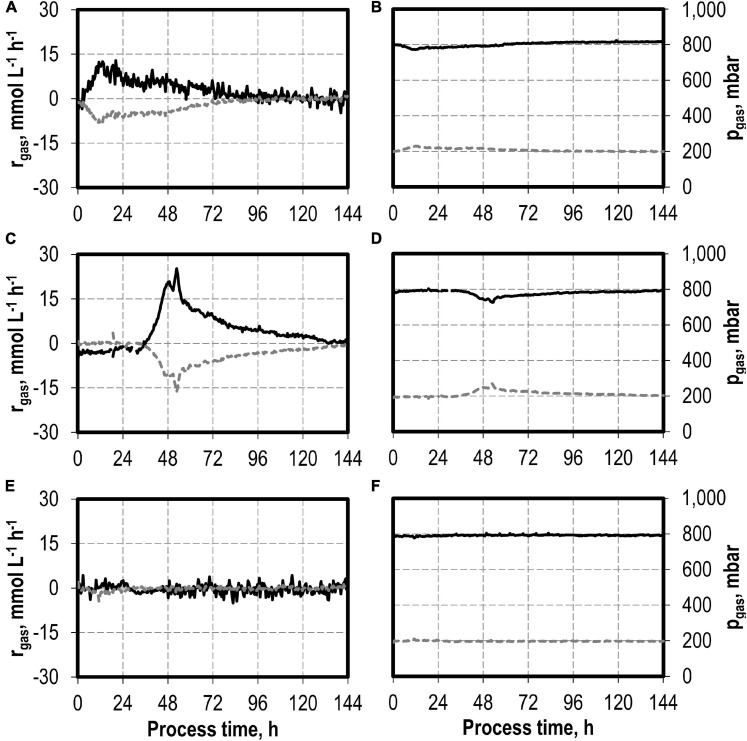
Gas consumption rates and gas partial pressures of CO (black line) and CO_2_ (dark gray dotted line) for autotrophic batch processes in continuously gassed stirred-tank bioreactors with *C. carboxidivorans* (*p*_CO_ = 800 mbar, *p*_CO__2_ = 200 mbar, *F*_gas_ = 0.083 L L^−1^ min^−1^, *T* = 37°C, P V^−1^ = 15.1 W L^−1^, pH_ini_ = 6.0, no pH control below pH 6.0 but one-sided control above pH 6.1) with no addition of NaNO_3_ **(A,B)**, initial addition of 0.1 g L^−1^ NaNO_3_ **(C,D),** and initial addition of 1.0 g L^−1^ NaNO_3_ **(E,F)**—no data after 66 h due to a failure of the μ-GC used for online gas analysis.

CO consumption and CO_2_ production rates were highest after the initial addition of 0.1 g L^−1^ NaNO_3_ (Figure 6). The maximum CO consumption rate of 28.3 mmol CO L^−1^ h^−1^ was increased by a factor of 2.2 compared to the reference batch process, but initiation of CO consumption (and CO_2_ production) was delayed. The total CO consumption and CO_2_ production of *C. carboxidivorans* in the 1-L scale stirred-tank bioreactor with the initial addition of 0.1 g L^−1^ NaNO_3_ were increased by 53 and 49%, respectively, compared to the reference process (Table 3). Thereby the molar CO/CO_2_ ratio was increased to 1.45 compared to the reference process (1.33) and the batch process with the initial addition of 0.1 g L^−1^ NaNO_3_ (1.41). Carbon recovery varies between 91 and 95%.

**TABLE 3:** Total consumption of CO and production of CO_2_, biomass, and products on C-mol basis and carbon balances, as well as maximum CDW concentrations, maximum product concentrations, and the maximum specific growth rate of the autotrophic batch processes with *C. carboxidivorans* in continuously gassed stirred-tank bioreactors (*V* = 1 L) with the initial addition of NaNO_3_.

	Reference process	+0.1 g L^−1^ NaNO_3_	+1.0 g L^−1^ NaNO_3_
Carbon in medium, mmol C	9.94	9.94	9.94
CO consumption, mmol C	436.69 ± 5.7	666.05	<50.00
CO_2_ production, mmol C	328.11 ± 14.5	473.95	<20.00
CO:CO_2_ ratio, –	1.33 ± 0.06	1.41	–
Carbon in products, mmol C	81.06 ± 1.03	146.10	11.54
Carbon in biomass, mmol C	13.98 ± 0.34	24.37	3.03
Carbon-balance (recovery), %	95 ± 3.0%	95%	–
CDW_max_, g L^−1^	0.40 ± 0.04	0.64	0.09
c_Acetate, max_, g L^−1^	0.96 ± 0.03	0.90	0.33
c_Ethanol, max_, g L^−1^	1.17 ± 0.18	0.88	0.23
c_Butyrat, max_, g L^−1^	0.21 ± 0.04	2.00	0.03
c_1__–Butanol, max_, g L^−1^	0.56 ± 0.10	0.03	0.01
c_Hexanoate, max_, g L^−1^	0.08 ± 0.01	0.00	0.00
c_1__–Hexanol, max_, g L^−1^	0.16 ± 0.04	0.00	0.00
μ_max_, h^−1^	0.116 ± 0.008	0.112	–

*Standard deviations are estimated based on the results of four individual reference batch processes; other batch processes were performed once.*

C-balances are not fully closed because non-consumed organic carbon in medium (yeast extract and cystein) and evaporation of the alcohols produced were not considered.

### Autotrophic Batch Processes With Varying Nitrite Additions

**FIGURE 7:**
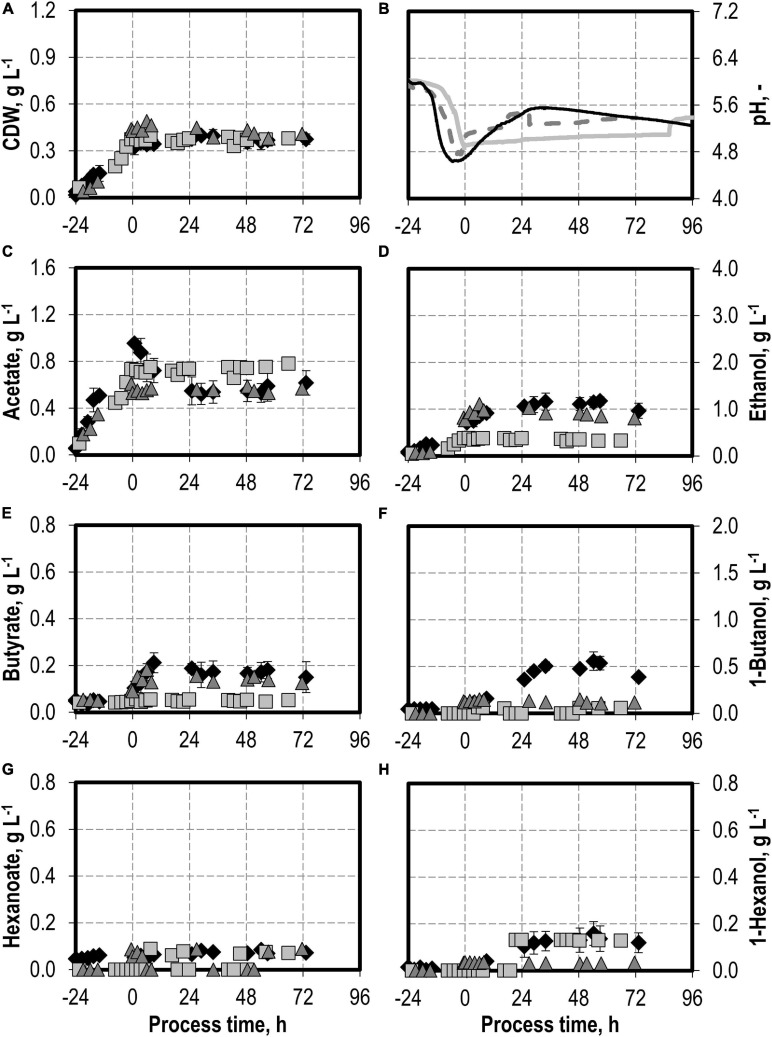
Autotrophic batch processes in continuously gassed stirred-tank bioreactors with *C. carboxidivorans* (*p*_CO_ = 800 mbar, *p*_CO__2_ = 200 mbar, *F*_gas_ = 0.083 L L^−1^ min^−1^, *T* = 37°C, P V^−1^ = 15.1 W L^−1^, pH_ini_ = 6.0, no pH control) with no addition of NaNO_2_ (reference process, black diamond, black line), addition of 0.1 g L^−1^ NaNO_2_ (gray square, gray solid line), and addition of 0.5 g L^−1^ NaNO_2_ (gray triangle, gray dashed line) after *C. carboxidivorans* concentrations of at least 0.3 g L^−1^ CDW were observed, indicated by a process time of 0 h. Error bars indicate the standard deviation of four reference processes**;** other batch processes were performed once.

**FIGURE 8:**
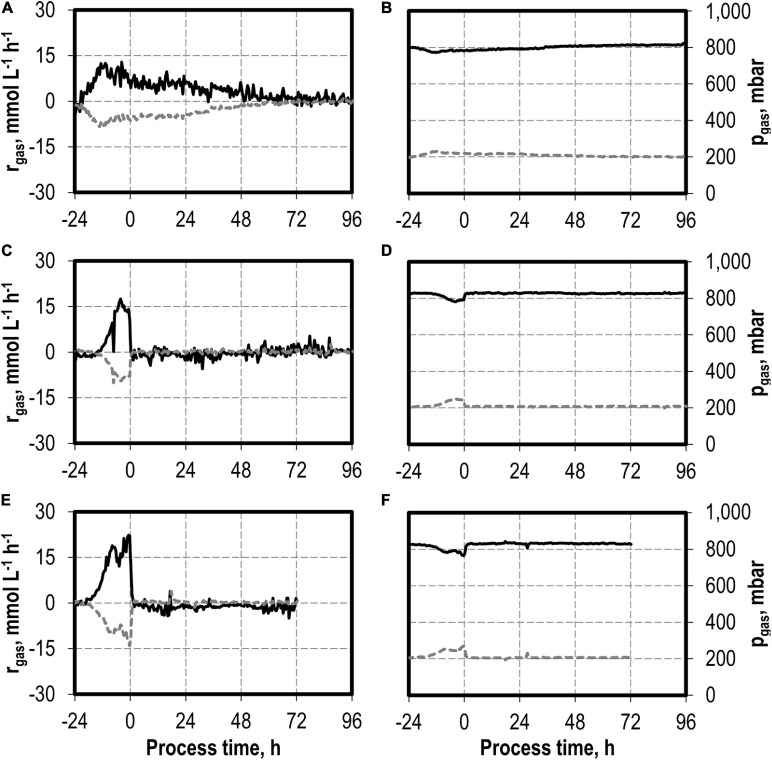
Gas consumption rates and gas partial pressures of CO (black line) and CO_2_ (dark gray dotted line) for autotrophic batch processes in continuously gassed stirred-tank bioreactors with *C. carboxidivorans* (*p*_CO_ = 800 mbar, *p*_CO__2_ = 200 mbar, *F*_gas_ = 0.083 L L^−1^ min^−1^, T = 37°C, P V^−1^ = 15.1 W L^−1^, pH_ini_ = 6.0, no pH control) with no addition of NaNO_2_ **(A,B)**, addition of 0.1 g L^−1^ NaNO_2_ **(C,D)**, and addition of 0.5 g L^−1^ NaNO_2_ **(E,F)**. The process time 0 h marks the injection of NaNO_2_ after *C. carboxidivorans* concentrations of at least 0.3 g L^−1^ CDW were observed, indicated by a process time of 0 h.

The addition of nitrite was carried out after *C. carboxidivorans* concentrations of at least 0.3 g L^−1^ CDW were reached in the stirred-tank bioreactors, indicated by a process time of 0 h in Figures 7, 8. CO consumption and CO_2_ production immediately stopped after nitrite addition, independently of the concentrations applied (0.1–0.5 g L^−1^ NaNO_2_). No further product formation was observed after nitrite additions. The nitrite concentrations tested are toxic for the metabolism of *C. carboxidivorans*, but the microorganism itself remains stable because the final biomass concentrations were not reduced compared to the reference process (Figure 7A).

## Discussion

The initial addition of up to 7.5 g L^−1^ NH_4_Cl to syngas batch fermentation processes with *C. carboxidivorans* resulted in up to 100% increased CO consumption, biomass formation, and the formation of alcohols (ethanol, butanol, and hexanol) with reduced production of organic acids compared to the reference batch process. Ammonia serves as a nitrogen source for *C. carboxidivorans* and thus boosts batch process performance if additionally supplied by the syngas. The typical amounts of NH_3_ in real synthesis gas of 4,500 ppm NH_3_ (Kremling et al., 2017; Kremling, 2018) would result in a maximum dissolved NH_3_ concentration of 0.133 mol L^−1^ NH_3_ in batch processes after 144 h, which corresponds to 7.1 g L^−1^ NH_4_Cl at the gas flow rate of 0.083 L L^−1^ min^−1^ applied in our fermentation studies assuming 100% absorption in the fermentation medium without microbial consumption. As a consequence, typical NH_3_ concentrations in real syngas will have no negative effect but may improve syngas batch fermentation processes with *C. carboxidivorans*.

In contrast to *C. carboxidivorans*, *C. ragsdalei* showed no additional ammonia consumption in batch processes applying syngas with 4,000 ppm NH_3_ at flow rates of 0.067 L L^−1^ min^−1^. It was reported that ammonium accumulated to 0.226 mol L^−1^ NH_3_ (12.1 g L^−1^ NH_4_Cl) already after 37 h (Xu et al., 2011). It is important to point out that the reported data on ammonia accumulation should be considered carefully because a simple mass balance assuming ideal gas behavior of all the syngas components at 37°C and 1 bar should have resulted in an accumulation of just 0.023 mol L^−1^ NH_3_ at the maximum.

The initial addition of up to 1.0 g L^−1^ H_2_S (2.2 g L^−1^ thioacetamide) to autotrophic syngas fermentation processes with *C. carboxidivorans* was beneficial for biomass formation (increase by a factor of 2.1) and alcohol production (increase by a factor of 2.7 for ethanol) compared to the reference batch process. An addition of 2.0 g L^−1^ H_2_S increased biomass growth and product formation as well, but prolonged the initial lag phase in the batch process. H_2_S serves as a possible sulfur source for *C. carboxidivorans* and acts as a reducing agent, and thus improves the process performance if additionally supplied by the syngas. 500 ppm H_2_S, a usual concentration in real syngas (Kremling et al., 2017; Kremling, 2018), corresponds to 0.33 g L^−1^ H_2_S at the maximum after 144 h in batch processes at a gas flow of 0.083 L L^−1^ min^−1^, assuming full absorption of H_2_S in the fermentation medium without microbial consumption applying the Henry constant for H_2_S (3.1 bar mol^−1^ in pure water) at 310 K and 1 bar (Suleimenov and Krupp, 1994). Stripping of H_2_S was not observed within the lower detection limit of the μGC instrument applied for online gas analysis (50 ppm H_2_S). As a consequence, typical H_2_S concentrations in real syngas will have no negative effect but may improve syngas batch fermentation processes with *C. carboxidivorans.*

Pure NO in syngas does not react with water but can support the reaction of NO_2_ supplied by syngas. NO_2_ disproportionates in water into HNO_2_ and HNO_3_ (Lee and Schwartz, 1981; Joshi et al., 1985). Up to 2 mol HNO_2_ is formed in water at equimolar concentrations of NO and NO_2_ (Joshi et al., 1985). At acidic pH, HNO_2_ is unstable and decomposes into HNO_3_ and NO (Chan et al., 1976; Joshi et al., 1985). A real syngas from gasification of biogenic residues typically contains around 200 ppm NO*_x_* (Kremling et al., 2017; Kremling, 2018). This would result in a maximum of 0.006 mol L^−1^ NO_2_^–^ (0.41 g L^−1^ NaNO_2_) after 144 h at a gas flow of 0.083 L L^−1^ min^−1^, assuming 100% absorption and no consumption by the bacteria. Assuming a total conversion of the NO*_x_* into NO_3_^–^ would result in a maximum of 0.006 mol L^−1^ NO_3_^–^ (0.55 g L^−1^ NaNO_3_).

The initial addition of a nitrate concentration as low as 0.1 g L^−1^ NaNO_3_ to autotrophic syngas fermentation processes with *C. carboxidivorans* increased the lag phase in the batch process and reduced the alcohol production at the expense of an increased formation of organic acids. Nitrate can be used as an alternative terminal electron acceptor by *Clostridia* (Emerson et al., 2019; Klask et al., 2020). The corresponding ammonium formation increases the pH in the fermentation broth, which resulted in initial H_2_SO_4_ addition for pH control. However, CO was not consumed in parallel, indicating the preference of *C. carboxidivorans* for using NO_3_^–^ before CO as the terminal electron acceptor. During nitrate reduction, yeast extract (1 g L^−1^) and cysteine (0.4 g L^−1^) provided initially with the fermentation medium may serve as carbon source for growth and product formation as no CO_2_ consumption was measured. The suppression of CO consumption in the presence of nitrate may be caused by the reported inhibition of the hydrogenase of *Clostridia* by nitric oxide species (Ahmed and Lewis, 2007) and the reduction in alcohol formation may be caused by the reported inhibition of the alcohol dehydrogenase by nitric oxide species (Gergel and Cederbaum, 1996). In contrast to *C. carboxidivorans, C. ljungdahlii* benefits from the initial addition of 15 mM NO_3_^–^ (1.3 g L^−1^ NaNO_3_) in autotrophic batch processes in anaerobic flasks with H_2_ and CO_2_, resulting in 75% higher growth rate and increased alcohol-to-acid ratio (Emerson et al., 2019). Other spore-forming *Clostridia* like *Clostridium perfringens* were reported to show a drastically reduced sporulation in the presence of different nitrate salts. Addition of 0.1 g L^−1^ Ca(NO_3_)_2_ led to a reduction of bacterial growth and of the number of spores by two orders of magnitude (Yasugi et al., 2016). It was also found that both nitrate and nitrite reductase were upregulated in the presence of nitrate salts. The authors concluded that the formation of toxic nitrite by reduction of nitrate might be responsible for the negative effects on growth and sporulation (Yasugi et al., 2016).

The addition of low concentrations of nitrite (0.1 and 0.5 g L^−1^ NaNO_2_, respectively) to autotrophic syngas fermentation processes with *C. carboxidivorans* after 0.3 g L^−1^ CDW has been produced in the batch processes resulted in an immediate truncation of CO consumption and any other metabolic activity, although *C. carboxidivorans* has a gene coding for a nitrite reductase in its genome (Li et al., 2015). Other anaerobic bacteria like *M. thermoacetica* are able to grow in the presence of up to 0.35 g L^−1^ NaNO_2_ (5 mmol L^−1^ NO_2_^–^) using nitrite as a terminal electron acceptor (Seifritz et al., 2003).

As a consequence, NO*_x_* concentrations in real syngas will have negative effects on syngas fermentation processes with *C. carboxidivorans.* Nitrite is toxic to *C. carboxidivorans* at the concentrations studied, and CO will not be used as terminal electron acceptor as long as nitrate is available in the aqueous phase (a threshold was not identified in this study). Thus, the purification of the syngas from NO*_x_* is a crucial aspect of the conception of a gas purification procedure for syngas fermentation processes with *C. carboxidivorans*.

## Conclusion

Syngas fermentation is a promising approach for sustainable production of biofuels or platform chemicals. However, the feasibility of the process is closely related to the usability of a real syngas from industry or from gasification of biogenic residues. In this study, effects of defined trace impurities from typical real syngas from gasification of biogenic residues were analyzed and, thus, quantitative criteria for gas purification could be identified. While syngas impurities like NH_3_ and H_2_S improved growth, alcohol formation, and CO consumption of *C. carboxidivorans*, NO*_X_* impurities largely decreased growth and led to the unwanted accumulation of organic acids and reduced alcohol formation. The findings of this study might play a crucial role in the technical realization of the coupling of gasification of biogenic residues, syngas purification, and syngas fermentation. The influence of combined trace impurities has not yet been fully investigated and can be a promising field for future research. The discovered effects of individual trace impurities in this work could lead to different interactions in the presence of combinations of these impurities. Therefore, studies on these effects could give an even closer insight on process performance with a real syngas.

## Data Availability Statement

The original contributions presented in the study are included in the article/Supplementary Material, further inquiries can be directed to the corresponding author/s.

## Author Contributions

AR and DW-B: conceptualization. AR, JH, CM, and AF: methodology and investigation. AR, JH, CM, AF, and DW-B: data discussion and analysis. AR: writing—original draft preparation and visualization. DW-B: writing—review and editing, supervision, project administration, and funding acquisition. All authors read and agreed to the published version of the manuscript.

## Conflict of Interest

The authors declare that the research was conducted in the absence of any commercial or financial relationships that could be construed as a potential conflict of interest.
