# Supplementary material for: Studies on Syngas Fermentation With Clostridium carboxidivorans in Stirred-Tank Reactors With Defined Gas Impurities
Source: Front Microbiol. 2021 Apr 15;12:655390. doi: 10.3389/fmicb.2021.655390 (PMC8081853; doi:10.3389/fmicb.2021.655390)
Supplement: Supplementary file 1 [file Table_1.DOCX]

Supplementary Material

**Table S1**: Medium (Doll et al., 2018) used for precultures in anaerobic shaken bottles and batch processes in stirred-tank bioreactors.

| Component | Formula | Concentration in Stock solution, g L^‑1^ |
| --- | --- | --- |
| Mineral Solution |  | **33.3x** |
| ammoniumchloride | NH_4_Cl | 100 |
| natrium chloride | NaCl | 80 |
| potassium chloride | KCl | 10 |
| potassium dihydrogen phosphate | KH_2_PO_4_ | 10 |
| magnesium sulfate | MgSO_4_ | 20 |
| Calcium chloride | CaCl_2_ | 4 |
|  |  |  |
| Vitamin solution |  | **100x** |
| pyridoxine | C_8_H_11_NO_3_ | 0.01 |
| thiamine | C_12_H_17_ClN_4_OS | 0.005 |
| riboflavine | C_17_H_20_N_4_O_6_ | 0.005 |
| calcium pantothenate | Ca(C_9_H_16_NO_5_)_2_ | 0.005 |
| liponic acid | C_8_H_14_O_2_S_2_ | 0.005 |
| para amino benzoic acid | C_7_H_7_NO_2_ | 0.005 |
| nicotinic acid | C_6_H_5_NO_2_ | 0.005 |
| Vitamin B12 | C_72_H_100_CoN_18_O_17_P | 0.005 |
| D-biotine | C_10_H_16_N_2_O_3_S | 0.002 |
| folic acid | C_19_H_19_N_7_O_6_ | 0.002 |
| 2 mercapto ethane sulfonic acid | C_2_H_6_O_3_S_2_ | 0.02 |
|  |  |  |
| Trace element solution |  | **100x** |
| Nitrilotriacetic acid | C_6_H_9_NO_6_ | 2.00 |
| Mangan sulfate | MnSO_4_ | 1.00 |
| Ammonium iron sulfate | NH_4_Fe(SO_4_)_2_ | 0.80 |
| cobalt chloride | CoCl_2_ | 0.20 |
| zinc sulfate | ZnSO_4_ | 0.20 |
| copper chloride | CuCl_2_ | 0.02 |
| nickel chloride | NiCl_2_ | 0.02 |
| sodium molybdate | Na_2_MoO_4_ | 0.02 |
| sodium selenate | Na_2_SeO_4_ | 0.02 |
| sodium wolframate | Na_2_WO_4_ | 0.02 |
|  |  |  |
|  |  | **medium concentration** |
| yeast extract |  | 1.0 g L^-1­^ |
| cysteine hydrochloride^a)^ | C_3_H_7_NO_2_S HCl | 0.4 g L^-1^ |
| morpholino ethane sulfonic acid^b)^ | C_6_H_13_NO_4_S | 15.0 g L^-1^ |

^a)^ not used for heterotrophic preculture in anaerobic shaken bottles

^b)^ only used for heterotrophic preculture in anaerobic shaken bottles
